# Ethical Considerations for Unblinding and Vaccinating COVID-19 Vaccine Trial Placebo Group Participants

**DOI:** 10.3389/fpubh.2021.702960

**Published:** 2021-06-24

**Authors:** Jenna Rose Stoehr, Alireza Hamidian Jahromi, Clayton Thomason

**Affiliations:** ^1^Feinberg School of Medicine, Northwestern University, Chicago, IL, United States; ^2^Plastic and Reconstructive Surgery Department, Rush University Medical Center, Chicago, IL, United States; ^3^The Center for Gender Confirmation Surgery, Weiss Memorial Hospital, The University of Illinois at Chicago, Chicago, IL, United States; ^4^Department of Religion, Health and Human Values, Rush University Medical Center, Chicago, IL, United States

**Keywords:** COVID-19, placebo, clinical trials, vaccine, vaccine ethics

## Introduction

The Pfizer-BioNTech, Moderna, and Johnson & Johnson COVID-19 vaccinations have been approved under “Emergency Use Authorization” by the United States Food and Drug Administration (FDA). Other vaccines, such as the Sputnik V and AstraZeneca vaccines, have begun to be distributed in other nations around the world after the publication of promising efficacy results. Multiple more vaccine candidates are likely to follow, which will still require safety and efficacy testing. As vaccines have been distributed in a tiered fashion to the public, there has been discussion and disagreement regarding the matter of vaccination of placebo groups from the past or upcoming trials (1). It has been argued that only trial participants (placebo group) who would be otherwise offered the vaccine outside of the trial [i.e., high risk participants or healthcare workers (HCWs)] should be unblinded and given the vaccine, while all other participants should remain blinded (2, 3). We argue that, once proven efficacious, vaccine makers and researchers have an ethical obligation to unblind the placebo groups of COVID-19 vaccine trials and offer them vaccine, based on the four principles of medical ethics.

## Non-maleficence and Autonomy

The first two principles to consider are non-maleficence and autonomy. The blinded placebo group is at increased risk of COVID-19 due to two main factors: participant behavior changes and the accelerated spread and morbidity of COVID-19. The first factor is related to non-maleficence, while the second invokes the principle of autonomy. Non-maleficence, or the obligation to not cause harm, must be considered, as keeping placebo groups blinded may put them at a higher risk of harm. As part of a blinded study, participants were likely informed that they should maintain all of the same safety precautions as if they are not vaccinated. However, participants who received placebo may change their behavior, either intentionally or unintentionally, and relax precautions as a result of the published efficacy results of the vaccines. This issue could be further compounded by the constantly changing guidelines for vaccinated and unvaccinated persons by federal agencies such as the US Centers for Disease Control and Prevention (CDC) and a lack of additional guidance from trial investigators. Previous research has demonstrated that humans change their short-term behavior to interact with more people after receiving a vaccine that is known to be effective (4). It was found that in the 48 h after receiving a flu vaccine, the average number of people with which study participants interacted doubled in comparison to their interactions in the previous 48 h. While the authors speculate that this effect may be due to viral antigen exposure, they also hypothesize that it could be due to the feeling of protection or invincibility elicited by vaccination. As an additional concerning factor, the development of virus variants in certain parts of the world with more aggressive transmissibility has further increased the potential harm of remaining unvaccinated (5). Thus, false reassurance and change of behavior could theoretically put placebo participants, who believe they were vaccinated, at a higher risk for contracting COVID-19, a harm which could be mitigated by unblinding them.

Second, the placebo group agreed to some risk when they consented to their involvement in the study. This is in accordance with the principle of autonomy, which requires that patients should fully understand the risks and benefits of any procedure prior to consent. It can be ethically acceptable to allow research participants to experience some risks in order to collect scientific or socially valuable data, even once a vaccine has been proven effective (1). However, in this case of the approved vaccines, the potential of this data collection is not more valuable than the risk to participants (i.e., acquiring COVID-19 infection and potentially death) and the public (i.e., an individual becomes a carrier and passes the virus to others) if participants are kept blinded. The risk of being unvaccinated has increased significantly since trials began: the spread of COVID-19 has overwhelmed the healthcare systems of many countries, the true morbidity of the virus has become more apparent, and increasingly transmissible variants have emerged. Therefore, the level of risk to which the participants originally agreed no longer applies, and the prior assessment of the incurred risks is no longer valid. Therefore, keeping the placebo group unblinded does not respect the principle of non-maleficence nor autonomy.

## Beneficence and Justice

The second set of principles to consider is beneficence and justice. The primary arguments for keeping the placebo group blinded include the ongoing collection of research data and public health gains, which draw upon beneficence, providing a treatment with the intention of doing good, and justice, the obligation for fair distribution of a treatment. We argue that vaccinating current placebo groups and strategically planning future trials can respect both principles to a greater extent.

The collection of long-term safety data is of paramount importance to ensure the safety and efficacy of the vaccines. However, there are already risks to the validity of ongoing data collection if trials continue as planned now that the efficacy data has been made public (6). Participants are beginning to drop out of trials if their status is not revealed, if they are antibody-negative, and/or they have the potential to be vaccinated through other means (7, 8). High-risk patients who do become eligible through other sources are likely to leave first, which could bias long-term safety and efficacy results. Investigators are also ethically bound by “Good Clinical Practice” guidelines to inform trial participants about information that may change their willingness to participate in the trial, that is, the published efficacy results and the availability of vaccine to the general public, which may lead to further drop-out rates (9). By being offered a vaccine when provided with this information, placebo participants would be more likely to remain enrolled in a trial, and additional long-term data could be collected by monitoring them for 1–2 years after they receive the vaccine. There would now be different cohorts of participants that received a vaccine at different times of year with exposure to virus variants, which may be able to provide helpful information about vaccine efficacy. There are multiple options available to continue to collect valuable research data in current trials while allowing placebo group vaccination, including conducting an “intent-to-continue” subgroup analysis, or adjusting to a crossover or open label design (2, 9). In addition, there may be an opportunity to recruit individuals who do not want to receive vaccine as a placebo group. While this will not allow for a true blinded and randomized comparison, it may still be possible to accrue valuable long-term data about vaccine efficacy through these suggested changes. In addition, other designs could be considered for new studies to be conducted in parallel, such as non-inferiority trials or human challenge trials (10). Vaccinating the placebo group maximizes individual and societal benefit, as it directly benefits the participants who receive vaccine and indirectly benefits the general population by keeping participants engaged in the trial and allowing for longer-term data collection even after vaccines have been preliminarily proved to be efficacious.

The second concern regarding public health gains appeals to both beneficence and justice, as it is argued that vaccine should be allocated in a tiered system in order of greatest need, which will overall improve public health. Those against vaccinating placebo groups have stated that vaccinating placebo group individuals (who are not front-line HCWs, elderly or individuals with comorbidities) would reduce public health gains (2). We agree that HCWs should be prioritized first in situations where there is a scarcity of vaccine. However, many countries have already been able to vaccinate most HCWs and are moving on to other tiers of distribution. Moreover, many individuals (both HCWs and members of the general public) do not intend to get the vaccine, and vaccine is at risk of going to waste (11, 12). In countries where vaccine is not yet available to the general public, we believe that the placebo group should be given priority in the next tier of vaccine distribution, above their respective risk group in the general population. In early 2021, both Pfizer and Moderna began to offer participants the option to become unblinded and receive the vaccine (13, 14). By vaccinating placebo groups publicly, we could further improve perception of vaccination in the public eye, which may potentially lead to greater vaccine acceptance and improved public health gains.

Undoubtedly, placebo groups are an important tool in evidence-based medicine. In certain circumstances, it may still be ethical to use a placebo group in COVID-19 vaccine trials. An expert group assembled by the World Health Organization (WHO) identified characteristics that establish when the use of a placebo group at the onset of a vaccine is acceptable (i.e., when no safe vaccine is available, and the vaccine will benefit the population in which it will be tested) and when it is not (i.e., when a safe and effective vaccine exists and is currently available, and risks to participants of delaying the vaccine cannot be mitigated) (15). In the case of COVID-19 vaccine trials, there is an opportunity to continue to run randomized controlled trials (RCTs) with placebo groups while simultaneously increasing the overall number of vaccinated persons by planning and executing vaccine trials in low- and middle-income countries (LMICs) that currently have limited or no vaccine access. Many LMICs have had limited access to currently approved COVID-19 vaccines, both due to cost and a lack of appropriate infrastructure to store and distribute vaccines (16, 17). By focusing on expanding trials of approved vaccines and experimental vaccines to LMICs, vaccine manufacturers and researchers will still be able to collect valuable data while providing the most good for the largest number of people. There are important ethical considerations to address while running vaccine trials in LMICs, namely ensuring that the placebo group is truly justified in that context and that local stakeholders are involved (15). As long as these issues are appropriately addressed, COVID-19 vaccine trials in LMICs may be conducted in accordance with the principles of both beneficence and justice.

## Conclusions

As COVID-19 vaccine trials continue and efficacy results are published, it will become increasingly more difficult and ethically fraught to maintain a valid placebo group, especially in high-income countries. By unblinding and vaccinating placebo participants regardless of distribution tier, researchers have the opportunity to address all four of the primary bioethical principles: beneficence, non-maleficence, autonomy, and justice. Consequently, the need for placebo groups may be satisfied in future trials in LMICs, which will allow for additional gains in the pursuit of beneficence, justice, and health for all.

## Author Contributions

All authors listed have made a substantial, direct and intellectual contribution to the work, and approved it for publication.

## Conflict of Interest

The authors declare that the research was conducted in the absence of any commercial or financial relationships that could be construed as a potential conflict of interest.

## References

[1] Wendler D Ochoa J Millum J Grady C Taylor HA. COVID-19 vaccine trial ethics once we have efficacious vaccines. Science. (2020) 370:1277–9. 10.1126/science.abf5084 33273060 PMC10107693

[2] Rid A Lipsitch M Miller FG. The ethics of continuing placebo in SARS-CoV-2 vaccine trials. JAMA. (2020) 325:219–20. 10.1001/jama.2020.25053 33315080

[3] Krause PR Fleming TR Longini IM Peto R Beral V Bhargava B. Placebo-controlled trials of covid-19 vaccines—why we still need them. N Engl J Med. (2021) 384:e2. 10.1056/NEJMp2033538 33264543

[4] Reiber C Shattuck EC Fiore S Alperin P Davis V Moore J. Change in human social behavior in response to a common vaccine. Ann Epidemiol. (2010) 20:729–33. 10.1016/j.annepidem.2010.06.014 20816312

[5] Lauring AS Hodcroft EB. Genetic variants of SARS-CoV-2-what do they mean? JAMA. (2021) 325:529–31. 10.1001/jama.2020.27124 33404586

[6] Wilson FP. COVID Vaccine Trial Placebo Group Deserves Priority Vaccination December 15th, 2020, (2020). Available online at: https://www.medscape.com/viewarticle/942576 (accessed January 8, 2021).

[7] Winkler R. Covid-19 vaccine studies may suffer as volunteers consider dropping out. Wall Street J. (2020).

[8] Rowland C. Elderly Begin to Drop Out of Novavax Vaccine Trial to Get Pfizer and Moderna Shots. Washington, DC: The Washington Post (2021).

[9] Dal-Re R Orenstein W Caplan AL. Trial participants' rights after authorisation of COVID-19 vaccines. Lancet Respir Med. (2021) 9:e30–e1. 10.1016/S2213-2600(21)00044-8 33476582 PMC7816575

[10] Dal-Ré R Caplan AL Gluud C Porcher. R. Ethical and scientific considerations regarding the early approval and deployment of a COVID-19 vaccine. Ann Intern Med. (2021) 174:258–60. 10.7326/M20-7357 33216636 PMC7713906

[11] Daly M Robinson E. Willingness to vaccinate against COVID-19 in the US: Longitudinal evidence from a nationally representative sample of adults from April-October 2020. Am J Prev Med. (2020). 10.1101/2020.11.27.20239970. [Epub ahead of print]. PMC7883746 33773862

[12] Guidry JPD Laestadius LI Vraga EK Miller CA Perrin PB Burton CW. Willingness to get the COVID-19 vaccine with and without emergency use authorization. Am J Infect Control. (2020) 49:137–42. 10.1016/j.ajic.2020.11.018 33227323 PMC7677682

[13] Covidvaccinestudy.com. (2021). Available online at: https://www.covidvaccinestudy.com/ (accessed March 1, 2021).

[14] Herper M. Pfizer and BioNTech speed up timeline for offering Covid-19 vaccine to placebo volunteers. STAT, (2021). Available online at: https://www.statnews.com/2021/01/01/pfizer-and-biontech-speed-up-timeline-for-offering-covid-19-to-placebo-volunteers/ (accessed January 1, 2021).

[15] Rid A Saxena A Baqui AH. Placebo use in vaccine trials: recommendations of a WHO expert panel. Vaccine. (2014) 32:4708–12. 10.1016/j.vaccine.2014.04.022 24768580 PMC4157320

[16] Mullard A. How COVID vaccines are being divvied up around the world. Nature. (2020). 10.1038/d41586-020-03370-6. [Epub ahead of print]. 33257891

[17] Kuehn BM. High-Income Countries Have Secured the Bulk of COVID-19 Vaccines. JAMA. (2021) 325:612. 10.1001/jama.2021.0189 33591354

